# ﻿Pollen morphology of *Clerodendrum* L. (Lamiaceae) from China and its systematic implications

**DOI:** 10.3897/phytokeys.235.111516

**Published:** 2023-11-10

**Authors:** Xiakai Huang, Rui Wu, Zheng Xiong, Zhonghui Ma

**Affiliations:** 1 Department of Agricultural College, Guangxi Key Laboratory of Sugarcane Biology, Guangxi University, Nanning 530004, Guangxi, China Guangxi University Nanning China

**Keywords:** *Clerodendrum*, pollen morphology, SEM, taxonomy

## Abstract

Pollen morphology of 26 taxa of *Clerodendrum*, as well as one species of *Volkameria* from China, was investigated through a scanning electron microscope (SEM). Pollen grains of *Clerodendrum* are monads, radiosymmetric and tricolpate, with medium or large size. The equatorial view of the pollen grains is spheroidal or subprolate and the polar view is (sub) circular or rounded triangular. The colpus membrane of the investigated taxa is sunken (rarely even). Five varying pollen types are delimited on the basis of exine sculpturing: (1) spine-tectum perforatum; (2) spine-tectum imperforatum; (3) microspine-tectum perforatum; (4) microspine-tectum imperforatum; and (5) obtuser spine. The results indicate that *Clerodendrum* is closely related to several genera in Lamiaceae, including *Aegiphila*, *Amasonia*, *Kalaharia*, *Tetraclea*, *Volkameria*, *Oxera*, *Faradaya*, and *Hosea*, as supported by previous phylogenic studies. Additionally, the conventional infrageneric classification of *Clerodendrum* based on inflorescence and leaf characters is not supported by the results. However, the palynological data can be used to identify some closely related species with similar external characteristics. In conclusion, the investigation of pollen morphology not only contributes novel data from palynology for *Clerodendrum* but also provides a basis for future comprehensive classification of this genus.

## ﻿Introduction

The genus *Clerodendrum* L. (Lamiaceae), comprising approximately 400 species, is mainly distributed in tropical and subtropical Asia, Africa, and America (Li et al. 2016). It belongs to the subfamily Ajugoideae (Harley et al. 2004), a monophyletic group divided into four main clades recognized as tribes: Ajugeae, Rotheceae, Teucrieae, and Clerodendreae. *Clerodendrum*, along with *Volkameria* L., *Kalaharia* Baill, *Amasonia* L.f., *Tetraclea* A. Gray, *Hosea* Ridl., *Aegiphila* Jacq., *Ovieda* L., and *Oxera* Labill., are members of Clerodendreae (Xiang et al. 2018; Zhao et al. 2021). However, generic relationships within Clerodendreae require further study (Zhao et al. 2021).

Previous infrageneric classification of *Clerodendrum* have been proposed using morphological characters. Based on inflorescence structure, Briquet (1895) divided the genus into three subgenera, whereas Thomas (1936) put forward a different classification relying on calyx structure. Moldenke (1985) merged the work of Thomas and Briquet, and sequentially raised a new classification with two large subgenera: *Tridens* Roem. & Schult. and *Volkameria*. Both Cantino (1992) and Rimpler et al. (1992) suggested that *Clerodendrum* may be paraphyletic or polyphyletic, but infrageneric relationships of the genus proposed by them were different. Based on molecular and morphological phylogenetic analyses, *Ovieda*, *Rotheca* Raf., and *Volkameria* were resurrected from *Clerodendrum* (Steane et al. 1997, 1999, 2004; Yuan et al. 2010).

In total, 34 species and 7 varieties of *Clerodendrum* are recorded from China and represented (Chen and Gilbert 1994). The most recent taxonomic treatment (Pei and Chen 1982) split the Chinese species into two sections based on corolla tube length and leaves’ arrangement. Section Siphonanthus Schauer comprised only one taxon that was characterized by corolla tube 5 cm long or longer, and leaves in whorls of 3–5, while section Clerodendrum included remaining species characterized by corolla tube less than 5 cm long, and opposite leaves or in whorls of three. However, throughout the field investigation and specimen examination, we have found that those quantitative characters are quite variable and can easily lead to misidentification. There is an urgent need to explore more morphological characters to build a more predictable classification system.

Palynological characters were considered as important characters for the taxonomy of Lamiaceae (Erdtman 1952; Abu-Asab and Cantino 1989, 1994; Abu-Asab 1991; Harley et al. 1992; Trudel and Morton 1992; Abu-Asab and Cantino 1994; Large and Mabberley 1995; Moon et al. 2008a, 2008b; Özler et al. 2011; Badamtsetseg et al. 2012; Ma et al. 2016). However, pollen morphology of the genus *Clerodendrum* is poorly known. Raj (1983) analyzed pollen morphology of 10 species of *Clerodendrum* using light microscope (LM), and only *C.bungei* Steud. was observed by scanning electron microscope (SEM). Perveen and Qaiser (2007) described the pollen structure of *C.phlomidis* L.f. in the palynology study of Verbenaceae in Pakistan. Liu (1985), in China, inspected the pollen morphology of 17 species and one variety of *Clerodendrum* comprising by light microscope, and only *C.cyrtophyllum* Turcz. was observed by SEM. Summarized from the studies mentioned above, it indicates that palynological characters such as variation in size, shape, and exine sculpturing are taxonomically valuable in the identification of closely related species in *Clerodendrum*.

In this study, we investigate the pollen morphology of 25 species and one variety of *Clerodendrum*, one species of *Volkameria* L. (*V.inermis* L.) which was traditionally placed within *Clerodendrum*. The objectives of this study are: (1) to provide extra palynological data to establish a more precise infrageneric classification for the genus; (2) further contribute to a comprehensive systematic study for *Clerodendrum* and clarify its relationship with other taxa of subfamily Ajugoideae.

## ﻿Materials and methods

Pollen grains of 26 taxa (Chen and Gilbert 1994) of *Clerodendrum* and one species *Volkameria* were sampled. Pollen material was mainly collected from herbarium specimens deposited at herbaria IBSC and KUN, and the rest were collected in the field (see Table 1).

**Table 1.** Specimens investigated.

Taxa	Collection localities	Collecting date	Collector	Number	Herbarium
*C.brachystemon* C.Y.Wu & R.C.Fang	Xizang, Motuo, 793 m	2019.8.24	Z. Xie	693	GAUA
*C.bracteatum* Wall. ex Walp.	Yunnan, Gongshan, 1889 m	1982.8.5	Gongshan Team	8889	KUN
*C.bungei* Steud.	Guangxi, Jinxiu, 820 m	1981.11.21	Dayaoshan Team	13341	IBSC
C.chinensevar.simplex (Moldenke) S. L. Chen	Guangxi, Baise, 646 m	2022.6.2	Z. Xiong et al.	JX001	GAUA
*C.colebrookianum* Walp.	Xizang, Motuo, 848 m	2019.8.24	Z. Xie	GXU0020	GAUA
*C.cyrtophyllum* Turcz.	Guangxi, Guigang, 110 m	2019.7.10	Z. H. Ma et al.	GXU0016	GAUA
*C.fortunatum* L.	Guangdong, Huizhou, 160 m	2013.9.07	H. G. Ye et al.	21973	IBSC
*C.garrettianum* Craib	Yunnan, 900 m	1936.9	C. W. Wong	78761	IBSC
*C.griffithianum* C.B.Clarke	Yunnan, Yingjiang,830 m	1981.2.24	S. W. Yu, Q. T. Zhang	602	KUN
*C.hainanense* Hand.-Mazz.	Hannan, Lingshui, 600 m	1956.10.30	L. Tang	2878	IBSC
*C.henryi* Pei	Guangdong, Guangzhou, 14 m	2021.5.23	R. Wu	GZ002	GAUA
*C.indicum* (L.) Kuntze.	Yunnan, Mengla, 580 m	2002.11.24	S. S. Zhou	570	IBSC
*C.japonicum* (Thunb.) Sweet	Guangdong, Guangzhou, 14 m	2021.5.23	R. Wu	GZ001	GAUA
*C.kaichianum* Hsu	Henan, Neixiang	2005.8.5	C. S. Zhu	2005095	IBSC
*C.kwangtungense* Hand.-Mazz.	Guangxi, Rongshui	1958.9.3	S. Q. Chen	16653	IBSC
*C.lindleyi* Decne. ex Planch.	Guangxi, Baise, 321 m	2019.10.12	Q. B. Zeng, Z. Xie	763	GAUA
*C.longilimbum* Pei	Yunnan Lingcang, 1500 m	1958.9.27	T. P. Zhu	0011	IBSC
*C.mandarinorum* Diels	Guangdong, Huaiji	2000.9	W. M. Yi, Z. F. Huang	15985	IBSC
*C.paniculatum* L.	Taiwan	1988.11.27	S. Z. Yang	11431	IBSC
*C.speciosum* Dombrain	Yunnan, Mengla, 570 m	2004.1.1	H. Wang.	7444	IBSC
*C.splendens* G. Don	Guangxi, Nanning, 79 m	2021.11.29	Z. Xiong	NN008	GAUA
*C.sylvestre* Moldenke	Guangxi, Guilin	1950.6.13	Z. S. Chung	808315	IBSC
*C.trichotomum* Thunb.	Hubei, Badong	1957.7.15	G. X. Fu	740	IBSC
C.trichotomumvar.fargesii (Dode) Rehder	Sichuan, Leibo, 1600 m	1989.8.13	Q. S. Zhao	517	IBSC
*C.villosum* Blume	Yunnan, Yingjiang	1986.11.18	86 Team	01033	KUN
*C.wallichii* Merr.	Yunnan, Mengla, 570 m	2004.3.22	H. wang	6427	IBSC
*V.inermis* L.	Guangdong, Lianjiang, -1.2 m	2019.10.1	Z. H. Ma et al.	750	GAUA

Flowers were dipped in glacial acetic acid (Reitsma 1969), and pollen was released using tweezers. Due to the friable nature of *Clerodendrum* pollen, the pollen samples for SEM were acetolysed for 10 min at indoor temperature that were adjusted from the method of Erdtman (1969). After acetolysis, pollen grains were progressively dehydrated in ethanol solutions of different concentrations (30%, 50%, and 70%) which were then bathed ultrasonically. Thereafter, the dehydrated pollen grains were dropped to a copper platform with double-side adhesive tape, air-dried and coated with gold. Pollen grains were observed and photographed using an electron microscope (Hitachi-S3400) at 10 kV. The measurements were based on randomly selected 20 pollen grains from each specimen using SEM, including the equatorial diameter (E), axis diameter (P) and the respective maximum, minimum and mean values.

The terminology used was according to Punt et al. (2007). Shape classes (P/E) were in accordance with Erdtman et al. (1993). Pollen size classes were plotted following Hesse et al. (2009). The pollen morphology of *Clerodendrum* is primarily described by qualitative and quantitative characteristics including shape, aperture type, colpus features and sexine ornamentation.

## ﻿Results

**Figure 1.**
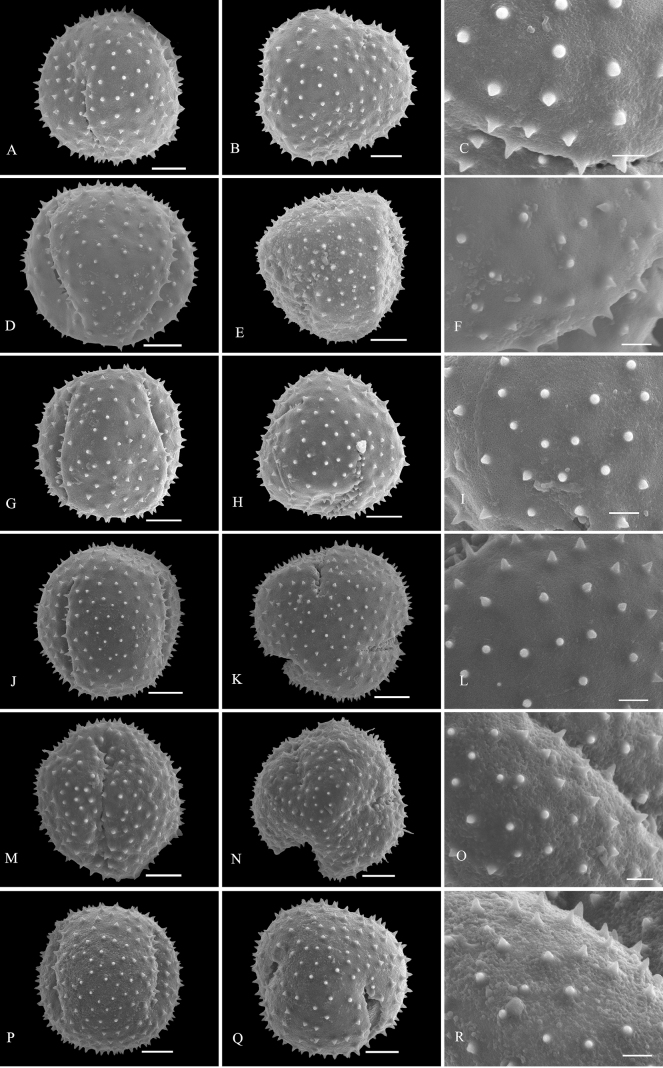
SEM micrographs of pollen grains of *Clerodendrum***A–C***C.chinense***D–F***C.villosum***G–I***C.trichotomum***J–L***C.mandarinorum***M–O***C.lindleyi***P–R***C.colebrookianum*. Equatorial view: **A, D, G, J, M, P**; polar view: **B, E, H, K, N, Q**; exine ornamentation: **C, F, I, L, O, R**. Scale bars: 10 μm (**A, B, D, E, G, H, J, K, M, N, P, Q**); 3 μm (**C, F, I, L, O, R**).

**Figure 2.**
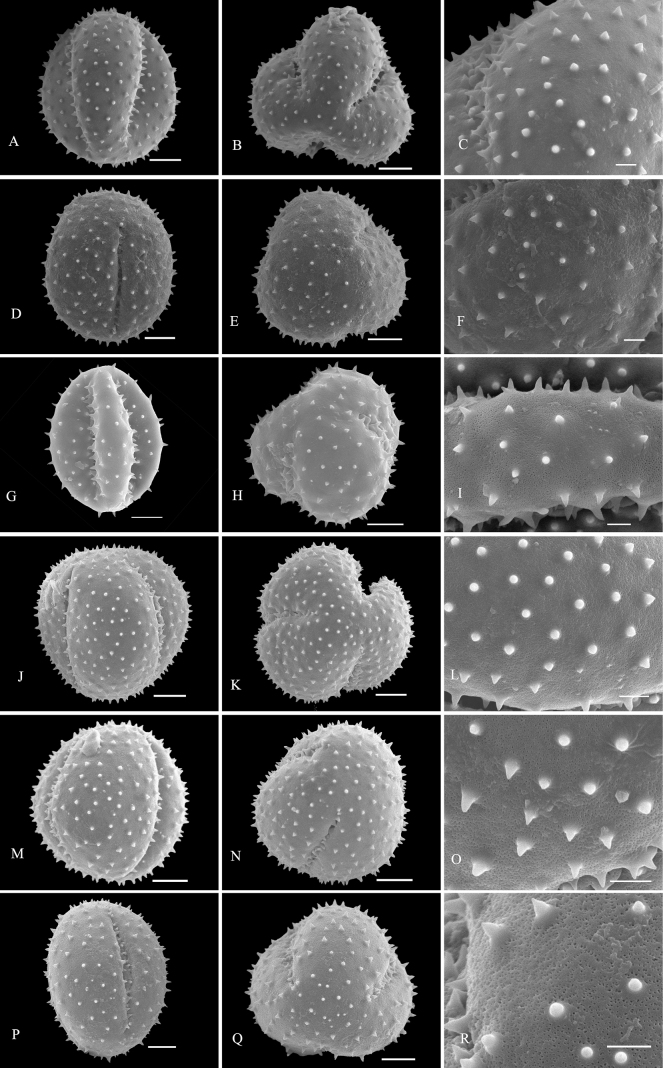
SEM micrographs of pollen grains of *Clerodendrum***A–C***Volkameriainermis***D–F**C.trichotomumvar.fargesii**G–I***C.bungei***J–L***C.hainanense***M–O***C.bracteatum***P–R***C.griffithianum*. Equatorial view: **A, D, G, J, M, P**; polar view: **B, E, H, K, N, Q**; exine ornamentation: **C, F, I, L, O, R**. Scale bars: 10 μm (**A, B, D, E, G, H, J, K, M, N, P, Q**); 3 μm (**C, F, I, L, O, R**).

**Figure 3.**
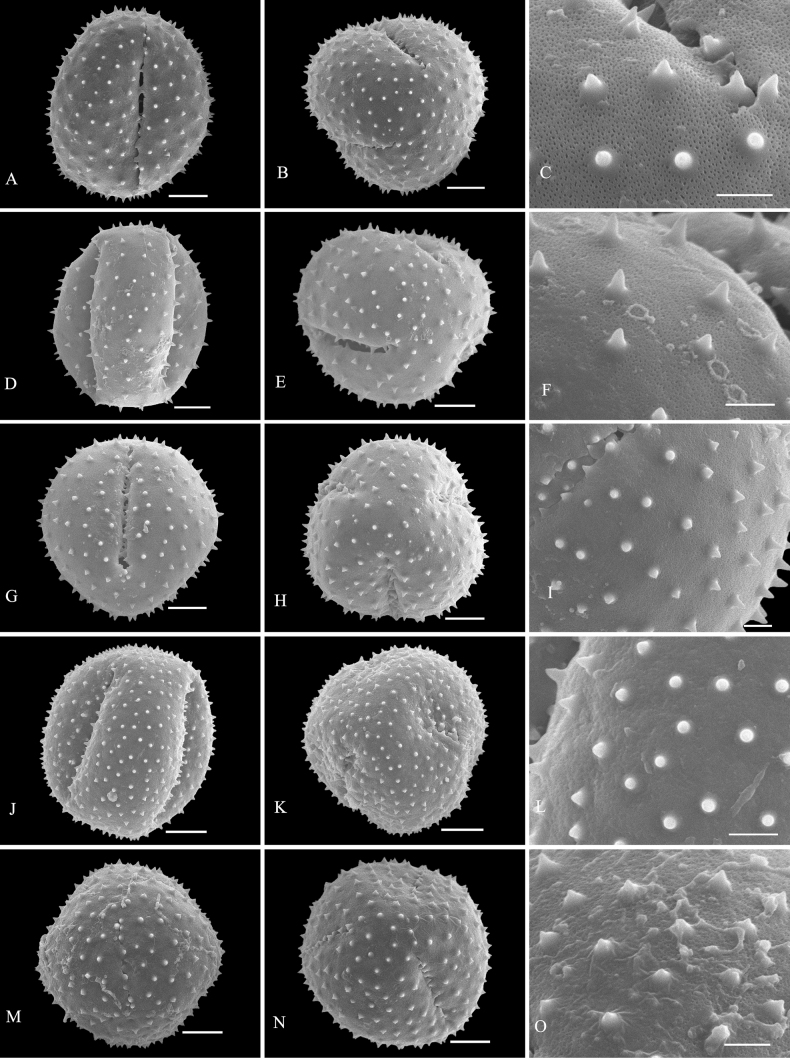
SEM micrographs of pollen grains of *Clerodendrum***A–C***C.brachystemon***D–F***C.kwangtungense***G–I***C.sylvestre***J–L***C.henryi***M–O***C.paniculatum*. Equatorial view: **A, D, G, J, M**; polar view: **B, E, H, K, N**; exine ornamentation: **C, F, I, L, O**. Scale bars: 10 μm (**A, B, D, E, G, H, J, K, M, N**); 3 μm (**C, F, I, L, O**).

**Figure 4.**
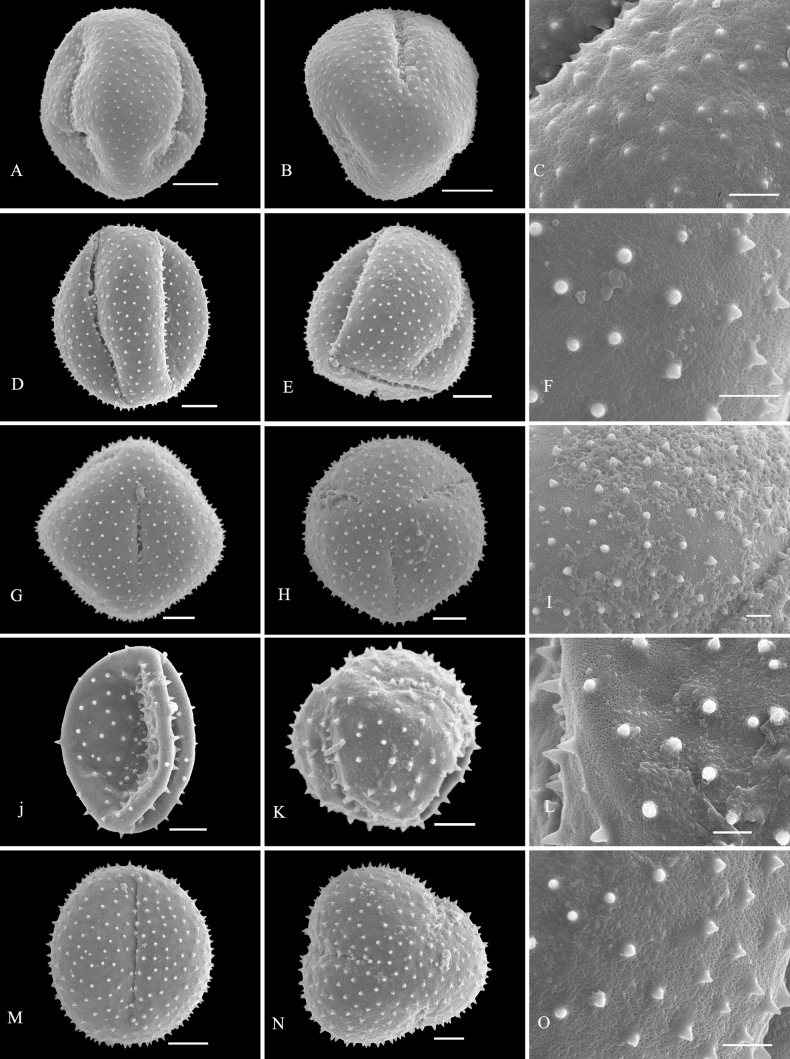
SEM micrographs of pollen grains of *Clerodendrum***A–C***C.fortunatum***D–F***C.garrettianum***G–I***C.japonicum***J–L***C.kaichianum***M–O***C.cyrtophyllum*. Equatorial view: **A, D, G, J, M**; polar view: **B, E, H, K, N**; exine ornamentation: **C, F, I, L, O**. Scale bars: 10 μm (**A, B, D, E, G, H, J, K, M, N**); 3 μm (**C, F, I, L, O**).

**Figure 5.**
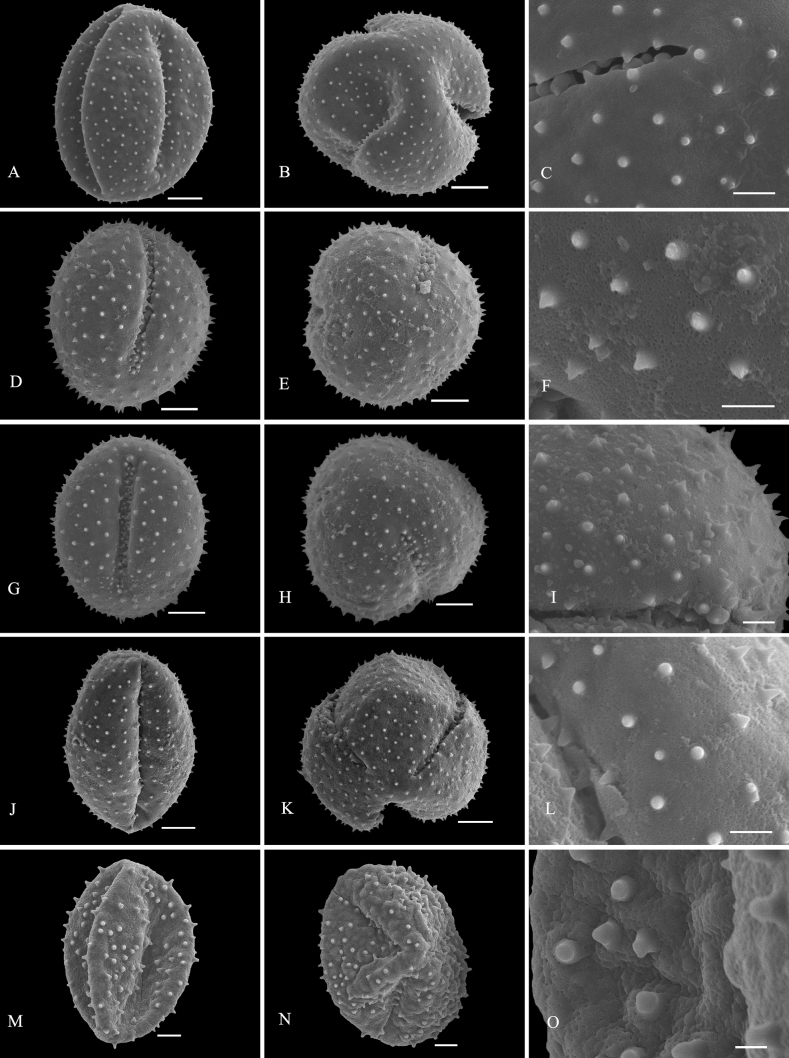
SEM micrographs of pollen grains of *Clerodendrum***A–C***C.longilimbum***D–F***C.speciosum***G–I***C.splendens***J–L***C.wallichii***M–O***C.indicum*. Equatorial view: **A, D, G, J, M**; polar view: **B, E, H, K, N**; exine ornamentation: **C, F, I, L, O**. Scale bars: 10 μm (**A, B, D, E, G, H, J, K, M, N**); 3 μm (**C, F, I, L, O**).

Palynological characteristics of all the investigated samples are given in Table 2 and illustrated in Figs 1–5.

**Table 2.** Pollen morphology of *Clerodendrum*.

Taxa	P value(μm)	E value(μm)	P/E	Size	Shape	Amb	Aperture type	Colpus membrane	Exine type	Figures
*C.brachystemon*	47.98 (44.44–52.93)	42.27 (37.40–46.12)	1.14	Medium	Subprolate	Rounded triangular	Tricolpate	Sunken	spine-tectum perforatum	3A–C
*C.bracteatum*	45.74 (40.95–52.14)	41.08 (36.52–43.93)	1.11	Medium	Spheroidal	Rounded triangular	Tricolpate	Sunken	spine-tectum perforatum	2M–O
*C.bungei*	48.92 (42.70–54.18)	36.20 (29.85–42.04)	1.35	Medium	Subprolate	Rounded triangular	Tricolpate	Sunken	spine-tectum perforatum	2G–I
*C.chinense*	51.59 (44.50–59.02)	40.76 (37.23–44.57)	1.27	Large	Subprolate	Subcircular	Tricolpate	Sunken	spine-tectum imperforatum	1A–C
*C.colebrookianum*	47.78 (42.03–53.44)	44.27 (40.72–49.44)	1.08	Medium	Spheroidal	Rounded triangular	Tricolpate	Sunken	spine-tectum imperforatum	1P–R
*C.cyrtophyllum*	42.27 (38.35–45.75)	40.65 (37.57–43.41)	1.04	Medium	Spheroidal	Rounded triangular	Tricolpate	Sunken	microspine-tectum perforatum	4M–O
*C.fortunatum*	47.37 (40.46–53.29)	36.16 (31.56–40.61)	1.31	Medium	Subprolate	Subcircular	Tricolpate	Sunken	microspine-tectum perforatum	4A–C
*C.garrettianum*	57.83 (49.63–65.89)	41.93 (36.11–46.69)	1.38	Large	Subprolate	Rounded triangular	Tricolpate	Sunken	microspine-tectum perforatum	4D–F
*C.griffithianum*	56.08 (49.86–64.19)	41.03 (36.42–44.96)	1.37	Large	Subprolate	Rounded triangular	Tricolpate	Sunken	spine-tectum perforatum	2P–R
*C.hainanense*	49.43 (43.11–56.79)	44.13 (35.78–49.33)	1.12	Medium	Spheroidal	Rounded triangular	Tricolpate	Sunken	spine-tectum perforatum	2J–L
*C.henryi*	46.54 (41.22–52.40)	40.10 (33.45–46.58)	1.16	Medium	Spheroidal	Circular	Tricolpate	Sunken	microspine-tectum imperforatum	3J–L
*C.indicum*	70.05 (45.45–83.76)	50.93 (38.68–59.63)	1.38	Large	Subprolate	Subcircular	Tricolpate	Sunken	obtuser spine	5M–O
*C.japonicum*	56.59 (52.75–63.39)	53.59 (49.43–58.10)	1.06	Large	Spheroidal	Circular	Tricolpate	Sunken	microspine-tectum perforatum	4G–I
*C.kaichianum*	49.21 (43.03–54.29)	37.05 (31.89–42.65)	1.33	Medium	Subprolate	Circular	Tricolpate	Sunken	microspine-tectum perforatum	4J–L
*C.kwangtungense*	48.20 (45.40–51.57)	42.80 (36.95–45.69)	1.13	Medium	Spheroidal	Rounded triangular	Tricolpate	Sunken	spine-tectum perforatum	3D–F
*C.lindleyi*	46.68 (39.81–53.88)	36.49 (32.23–39.91)	1.28	Medium	Subprolate	Rounded triangular	Tricolpate	Sunken	spine-tectum imperforatum	1M–O
*C.longilimbum*	53.75 (48.57–61.53)	42.66 (35.05–47.82)	1.26	Large	Subprolate	Rounded triangular	Tricolpate	Sunken	microspine-tectum perforatum	5A–C
*C.mandarinorum*	41.95 (39.77–45.27)	39.96 (37.51–42.30)	1.05	Medium	Spheroidal	Circular	Tricolpate	Sunken	spine-tectum imperforatum	1J–K
*C.paniculatum*	49.08 (44.87–56.81)	45.44 (38.50–50.13)	1.08	Medium	Spheroidal	Circular	Tricolpate	Sunken	microspine-tectum imperforatum	3M–O
*C.speciosum*	47.68 (44.18–50.53)	45.20 (41.77–50.77)	1.05	Medium	Spheroidal	Circular	Tricolpate	Sunken	microspine-tectum perforatum	5D–F
*C.splendens*	45.46 (38.36–51.00)	41.63 (38.95–46.02)	1.09	Medium	Spheroidal	Rounded triangular	Tricolpate	Sunken	microspine-tectum perforatum	5G–I
*C.sylvestre*	44.87 (42.39–49.00)	42.60 (38.17–45.67)	1.05	Medium	Spheroidal	Rounded triangular	Tricolpate	Sunken	spine-tectum perforatum	3G–I
*C.trichotomum*	42.81 (38.50–47.65)	40.89 (38.25–43.78)	1.05	Medium	Spheroidal	Subcircular	Tricolpate	Sunken	spine-tectum imperforatum	1G–I
C.trichotomumvar.fargesii	46.71 (43.60–49.45)	43.35 (40.45–44.92)	1.08	Medium	Spheroidal	Rounded triangular	Tricolpate	Sunken	spine-tectum perforatum	2D–F
*C.villosum*	44.95 (38.62–52.91)	38.24 (33.53–42.20)	1.18	Medium	Subprolate	Subcircular	Tricolpate	Sunken	spine-tectum imperforatum	1D–F
*C.wallichii*	51.26 (44.92–60.28)	39.62 (30.71–43.80)	1.29	Large	Subprolate	Rounded triangular	Tricolpate	Sunken	microspine-tectum perforatum	5J–L
*V.inermis*	55.31 (44.03–62.04)	42.02 (36.15–45.43)	1.32	Large	Subprolate	Rounded triangular	Tricolpate	Sunken	spine-tectum imperforatum	2A–C

Pollen grains of *Clerodendrum* (including *Volkameriainermis*) are radiosymmetric, tricolpate, and monads. The size of pollen grains is medium (26–50 μm) or large (50–100 μm). The average value of the polar axis (P) is measured as 49.48 μm (41.95–70.05 μm) and that of equatorial diameter (E) is 41.89 μm (36.16–53.59 μm). The P/E ratio varies from 1.04 (*C.cyrtophyllum*) to 1.38 (*C.garrettianum* Craib). The pollen shape class is mainly spheroidal (0.88–1.14) or subprolate (1.14–1.33).

Exine sculpture can be divided into five different types: (1) spine-tectum perforatum, (2) spine-tectum imperforatum, (3) microspine-tectum perforatum, (4) microspine-tectum imperforatum, and (5) obtuser spine. The grains of type (1) are the largest groups, accounting for over a quarter of all the investigated species (Figs 2D–R, 3A–I). The type (2) with a rounded tectal perforation, less than 1 μm in diameter, can be distinguished from the spine-tectum perforatum (Figs 1A–R, 2A–C). Compared with type (1) and type (2), the type (3) (Figs 4A–O, 5A–L) and the type (4) (Fig. 3J–O) have more spines and are less than 1 μm in length. The type (5) is only found in one taxon (*C.indicum* (L.) Kuntze.) (Fig. 5M–O), which happens to be the only species of sect. Siphonanthus.

## ﻿Discussion

### ﻿Taxonomic implications of pollen morphology of *Clerodendrum*

Most pollen grains of *Clerodendrum* species investigated here are spheroidal or subprolate in equatorial view. Subprolate or prolate pollen grains observed in some species (*C.bungei*, *C.intermedium* Chamisso, *C.phlomidis*) and reported in previous studies (Raj 1983; Perveen and Qaiser 2007) were probably affected by the acetolysis treatment, as the pollen shape of Lamiaceae is easily affected during hydration and/or fixation (Sebsebe and Harley 1992). On account of the broken colpus membranes, oblate or suboblate pollen grains are easily incorrectly considered as subprolate or prolate (Harley 1992). Raj (1983) described pollen of *C.speciosissimum* C. Morren as distinct with three to four to six apertures, which was the first report of non-triaperturate pollen in *Clerodendrum* (without illustration). In our study, in contrast, only triaperturate pollens were observed from *Clerodendrum* (Figs 1–5). Based on the numbers of apertures and the characters of exine sculpture, Liu (1985) considered that pollen grains of most species of *Clerodendrum* (excepting *C.fortunatum* L., *C.paniculatum* L., *C.trichotomum* Thunb., and *C.yunnanense* Hu ex Hand.-Mazz.) are tricolpate-spiny, characterized by large or very large size, tricolpate, dense or sparse spine over the microreticulate exine ornamentation. Overall, our results are mostly consistent with the finding of Liu (1985). However, *C.fortunatum*, *C.paniculatum*, *C.trichotomum* and *C.yunnanense* pantocolpate pollen grains are as described by Liu (1985). This type is characterized by spheroidal grains of large size, with pantocolpate (6 to 8) and spiny exine. In contrast, all pollens grains of *Clerodendrum* (including *C.fortunatum*, *C.paniculatum* and *C.trichotomum*) observed in this study are tricolpate with dense or sparse spine in the exine (Figs 1–5).

The infrageneric classification system applied in Flora of China (Chen and Gilbert 1994) is not fully supported by our results. In comparison to the pollen shape and size, the exine sculpture appears to be a more taxonomically valuable and steady character. There is a clear distinction in pollen exine sculpture between sect. Siphonanthus and sect. Clerodendrum. The obtuser spine exine sculpture is only found in *C.indicum* (Fig. 5M–O), the sole species of sect. Siphonanthus. However, consistency between morphology and exine sculpture is hardly found in taxa belonging to sect. Clerodendrum. For example, species in Ser. Axilliflorae Schauer have different exine sculpture types: *C.griffithianum* C. B. Clarke is spine-tectum perforatum (Fig. 2P–R); *C.fortunatum* is microspine-tectum perforatum (Fig. 4A–C) and *Volkameriainermis* (=*C.inerme* (L.) Gaertn.) belongs to the spine-tectum imperforatum type (Fig. 2A–C). A similar phenomenon is observed in Ser. Densiflora Schauer: *C.bracteatum* Wall. ex Walp. and *C.bungei* belong to spine-tectum perforatum type, while *C.lindleyi* Decne. ex Planch. and C.chinensevar.simplex (Moldenke) S. L. Chen. belong to spine-tectum imperforatum type (Table 2); In Ser. Penduliflorae Schauer: *C.cyrtophyllum*, *C.garrettianum*, *C.wallichii* Merr. and *C.longilimbum* Pei belong to the microspine-tectum perforatum type, *C.hainanense* Hand.-Mazz. and *C.kwangtungense* Hand.-Mazz. belong to the spine-tectum perforatum type and *C.henryi* Pei belongs to microspine-tectum imperforatum type (Table 2); In Ser. Paniculata Schauer: *C.colebrookianum* Walp., *C.mandarinorum* Diels, *C.trichotomum* and *C.villosum* Blume belong to the spine-tectum imperforatum type (Table 2), *C.brachystemon* C. Y. Wu et R. C. Fang belongs to the spine-tectum perforatum type and *C.kaichianum* Hsu belongs to the microspine-tectum perforatum type (Table 2); In Ser. Squamata Schauer: *C.japonicum* (Thunb.) Sweet belongs to the microspine-tectum perforatum type (Table 2), whereas *C.paniculatum* belongs to the microspine-tectum imperforatum type (Table 2). In conclusion, the pollen morphology of Chinese *Clerodendrum* species investigated in this study does not provide obvious evidence for infra-sectional classification.

Pollen characteristics have been proven to be useful in species delimitation in some genera of Lamiaceae (Erdtman 1952; Abu-Asab and Cantino 1989, 1994; Abu-Asab 1991; Trudel and Morton 1992; Large and Mabberley 1995; Moon et al. 2008a, 2008b; Özler et al. 2011; Badamtsetseg et al. 2012; Ma et al. 2016). According to our results, the varieties could be easily distinguished from the original variety. For instance, the pollen tectum of *Clerodendrumtrichotomum* is imperforated, whereas that of C.trichotomumvar.fargesii (Dode) Rehder is perforated. In addition, species that are difficult to distinguish from one another based on external morphology can be discerned at the pollen level. For example, *C.wallichii* and *C.henryi* share many similarities such as 4-angled branchlets, white corolla, ovate lobes, exserted stamens, and style (Chen and Gilbert 1994). The two species are challenging to differentiate at first glance due to their overlapping habitats, similar plant height, and leaf shape. The primary identification characteristic relies on the length of the petiole: which in the case of *C.wallichii* is typically about 1 cm, while *C.henryi* generally exceeds 1 cm in length. Additionally, young branches of *C.wallichii* may exhibit winged branchlets, whereas this characteristic is absent in *C.henryi* (Chen and Gilbert 1994). Our study suggests that morphology of pollen grains can help to distinguish *C.wallichii* (with perforatum tectum; Fig. 5J–L), from *C.henryi* (imperforated tectum; Fig. 3J–L). Morphologically, *Clerodendrumlindleyi* is very similar to *C.bungei* and shares some characteristics: leaf blade broadly ovate to cordate; terminal inflorescences, dense, capitate, corymbose cymes; corolla pinkish to purple, lobes obovate; drupes blue-black and subglobose (Chen and Gilbert 1994). However, our study indicates that they are easily distinguished from each other because the exine sculpture type pollen of *C.bungei* has a perforated tectum (Fig. 2G–I), whereas that of *C.lindleyi* is imperforated (Fig. 1M–O).

### ﻿Comparison of pollen morphology of *Clerodendrum* and related genera within Lamiaceae

Pollen grains of Lamiaceae are commonly monad, isopolar, and there is a significant relationship between the number of pollen colpi in the subfamilies of Lamiaceae (Abu-Asab and Cantino 1989; Large and Mabberley 1995; Ma et al. 2016). The tectum of the pollen grains in Ajugoideae usually exhibits projections and spinules. For example, supratectal projections are conical in *Cardioteucris* C.Y. Wu and *Amethystea* L. (Abu-Asab and Cantino 1989); spinose in *Tripora* P.D. Cantino et al. (1998) and *Trichostema* Gronov. (Harley et al. 2004); spinulose in *Caryopteris* Bunge (Abu-Asab et al. 1993) and *Teucrium* L. (Harley et al. 2004). Perveen and Qaiser (2007) described the ornamentation of *Clerodendrumphlomidis* (*C.phlomidis*) as *Clerodendrumphlomidis*-type (Tectum reticulate with spinules or reticulate-rugulate). Our results complement these previous findings.

*Clerodendrum* is currently placed within Ajugoideae of Lamiaceae based on molecular phylogenetic evidence (Wagstaff and Olmstead 1997; Wagstaff et al. 1998). Recently, Zhao et al. (2021) further divided Ajugoideae into four tribes (Ajugeae, Clerodendreae, Teucrieae and Rotheceae) and assigned *Clerodendrum* and *Volkameria* in Clerodendreae. In our palynological study, the pollen grains of *V.inermis* (Fig. 2) are spine-tectum imperforatum, which is the same as found in several species of *Clerodendrum* (*C.villosum*, *C.trichotomum*, *C.mandarinorum*, *C.colebrookianum* and *C.lindleyi*; Fig. 1; Table 2). Therefore, our palynological data support the close relationship between *Clerodendrum* and *Volkameria* (Zhao et al. 2021). Barrabe et al. (2015) suggested that *Clerodendrum* has a close relationship with *Amasonia*, *Kalaharia*, *Tetraclea*, and *Volkameria*, and that they are sister taxa to the alliance encompassing *Hosea*, *Aegiphila*, *Oxera*, and *Faradaya*. Zhao et al. (2021) found that *Clerodendrum* was grouped together with *Oxera* and *Volkameria*. Here, pollen morphology supports Barrabe et al.’s (2015) and Zhao et al.’s (2021) findings. Palynological evidence has revealed that pollen characteristics of *Clerodendrum* (Figs 1–5; Table 2) and *Volkameria* (Fig. 2; Table 2), are similar to those of *Aegiphila*, *Amasonia*, *Faradaya*, *Hosea*, *Kalaharia*, *Tetraclea* and *Oxera* (Raj 1983). Most species exhibit radiosymmetric, tricolpate, monads, spiny ornamentation and spheroidal or subprolate shapes. Although palynological evidence supports that *Clerodendrum* is closely related to other eight genera, accurate relationships among those genera require a more comprehensive study.

## ﻿Conclusions

The pollen morphology of *Clerodendrum* from China was systematically reported for the first time in this study. Pollen morphology supports that *Clerodendrum* is a member of Ajugoideae, and some characteristics have significant taxonomic value for infraspecific classification and the identification of morphologically closely related taxa within *Clerodendrum*.

## ﻿Additional information

### Author ORCIDs

Xiakai Huang https://orcid.org/0000-0003-4515-5199

Rui Wu https://orcid.org/0000-0003-0484-3761

Zheng Xiong https://orcid.org/0000-0003-4295-2432

Zhonghui Ma https://orcid.org/0000-0002-3898-3079

### Data availability

All of the data that support the findings of this study are available in the main text.
